# Perceived stress and structural empowerment profiles among clinical nurses: a latent profile analysis in occupational health

**DOI:** 10.3389/fpubh.2026.1869234

**Published:** 2026-07-08

**Authors:** Wei Zhao, Fei Guo, Wen-Nv Hao

**Affiliations:** 1 Inner Mongolia Medical University, Hohhot, China; 2 The Affiliated Hospital of Inner Mongolia Medical University, Hohhot, China

**Keywords:** hospital nurses, latent profile analysis, occupational health, perceived stress, structural empowerment, workforce wellbeing

## Abstract

**Background:**

Healthcare workers, particularly nurses, face high occupational stress andinadequate organizational support, which threaten workforce stability and public health service quality. Structural empowerment is a core organizational resource for improving occupational health and wellbeing among nurses.

**Objective:**

To identify latent profiles of structural empowerment among clinical nurses, examine their distribution characteristics, and explore associations with perceived stress and occupational factors, to support evidence-based occupational health interventions and workforce management strategies.

**Methods:**

A cross-sectional survey was conducted among 518 clinical nurses from a tertiary general hospital in Inner Mongolia, China. We collected demographic data using a general information questionnaire. Structural empowerment was measured using the six-dimensional Conditions of Work Effectiveness Questionnaire-II, and perceived stress was assessed with the Perceived Stress Scale-10. Latent profile analysis was performed in Mplus8.3 to identify empowerment profiles, followed by pairwise binary logistic regression to examine the cross-sectional associations between perceived stress, demographic variables, and profile membership.

**Results:**

The mean score of structural empowerment among clinical nurses was (43.76 ± 12.93), and the mean score of perceived stress was (26.83 ± 7.92). The optimal latent profile model was a 4-class model: low structural empowerment group (66.6%, *n* = 345), moderate-low structural empowerment group (10.2%, *n* = 53), moderate structural empowerment group (11.4%, *n* = 59), and high structural empowerment group (11.8%, *n* = 61). Multivariate Logistic regression analysis showed that monthly night shift frequency in the past year, self-rated health status, involvement in or occurrence of nursing adverse events in the past year, and perceived stress were core influencing factors of structural empowerment.

**Conclusion:**

Structural empowerment among clinical nurses comprises four levels: low, moderate-low, moderate, and high. It is influenced by night shift frequency, self-rated health, nursing adverse events, and perceived stress. These findings support targeted interventions: optimizing shift scheduling, reducing work stress, prioritizing nurse health, and reframing adverse events as learning opportunities, to enhance nurse wellbeing, workforce stability, and public health service delivery.

## 1 Introduction

The World Health Organization (WHO) released the Global Strategic Directions for Nursing and Midwifery 2021–2025, emphasizing the release of nursing productivity through organizational empowerment and the improvement of nursing service efficiency via structural empowerment (1). Nursing is characterized by high technology, high risk, high work pressure, and high stressfulness (2). Nurses often face challenges such as resource shortages, time constraints, interpersonal pressure, and workplace bullying in practice (3). As an important incentive and supportive measure, empowerment has become a key strategy in nursing management. Structural empowerment refers to the ability of an organization to provide employees with resources needed to support their work, enabling employees to access work-related information, opportunities, support, and resources to achieve their work goals (4). In nursing practice, structural empowerment can create a positive organizational atmosphere, enhance nurses’ autonomy and sense of belonging, thereby better meeting patients’ needs and compensating for the deficiencies of traditional nursing management. Insufficient empowerment tends to reduce nurses’ motivation and impair nursing quality and patient safety. Perceived stress refers to an individual’s subjective evaluation of demands exceeding their coping capacity (5). Studies have shown a negative correlation between perceived stress and structural empowerment: higher perceived stress is associated with lower structural empowerment among clinical nurses (6).

Based on the Job Demands-Resources (JD-R) theory, structural empowerment is an important occupational resource that plays a vital role in improving job performance, reducing constraints, and enhancing nurses’ participation in decision-making (7). In nursing, high demands include frequent night shifts, exposure to adverse events, and heavy workloads, which consume physical and cognitive energy. Job resources, such as structural empowerment (i.e., access to information, support, resources, and opportunities), help nurses meet these demands and buffer their negative effects. Perceived stress is conceptualized as a psychological response to the imbalance between high demands and insufficient resources. Under high perceived stress, nurses’ cognitive resources and energy are largely consumed, making it difficult for them to effectively perceive and utilize empowerment resources provided by the organization (5). Therefore, this study elucidates the distribution of heterogeneous characteristics of structural empowerment among clinical nurses, which facilitates the identification of subgroups with distinct profiles and their associated determinants.

Latent Profile Analysis (LPA), a statistical method for classifying subjects based on latent variable characteristics, can effectively identify hidden homogeneous subgroups within a population and provide a new perspective for revealing the heterogeneity of individual behaviors (8). As a person-centered statistical method, LPA can detect latent subgroup structures in data and classify distinct latent profiles, thereby effectively reflecting the heterogeneity of study samples (9). Different from conventional variable-centered methods that only analyze overall scores and group averages, LPA is capable of identifying heterogeneous subgroups with unbalanced dimensional characteristics (10), which cannot be detected by traditional statistical approaches. This approach has been increasingly widely adopted in nursing research and has achieved favorable research outcomes (11). Most previous studies on structural empowerment among clinical nurses have adopted variable-centered approaches, which fail to identify individual differences within groups (6). Current studies on structural empowerment among clinical nurses mostly focus on analyzing influencing factors of the overall level (12, 13), lacking in-depth investigation of latent category distribution and category-specific determinants.

This study targeted clinical nurses to conduct latent profile analysis of structural empowerment, clarify the category characteristics and influencing factors of different empowerment subgroups, and provide empirical evidence to implement precise empowerment management, relieve nurses’ stress, optimize the work environment, and inform occupational health interventions and workforce policies to stabilize the nursing workforce and improve public health delivery.

## 2 Methodology

### 2.1 Research design

This study adopted a cross-sectional survey design and was implemented and reported in accordance with the Strengthening the Reporting of Observational Studies in Epidemiology (STROBE) reporting guidelines.

### 2.2 Research subjects and sampling

The research site was a tertiary grade A general hospital in the Inner Mongolia region. Questionnaires were distributed online through the Wenjuanxing^1^ platform using a convenient sampling method. The survey period was from January to March, 2026. Inclusion criteria were: registered nurses holding valid nursing practice certificates, having worked in clinical nursing for 1 year or more, and voluntarily participating and completing informed consent. Nursing interns, students in school, those on leave for more than 3 months (including maternity leave, sick leave, and training leave), as well as nurses from other institutions on training or rotation were excluded.

Sample size adequacy was evaluated against the requirements of the two analytic stages. Latent profile analysis with three or more well-separated classes is generally considered stable at sample sizes of three hundred or above (9). For the subsequent pairwise binary logistic regression, the events-per-variable rule of at least ten observations per predictor was satisfied for each pairwise comparison given the final set of retained predictors (14). Accounting for an anticipated invalid response rate of 10–20%, the final analytic sample of 518 clinical nurses satisfied these requirements.

### 2.3 Survey tools

#### 2.3.1 General information questionnaire

Seventeen items covering gender, age, marital status, highest educational background, professional title, position, employment method, work experience, department, average monthly income, monthly night shift frequency in the past year, average daily working hours, sleep status, self-rated health status, experience of verbal or physical violence from patients/families in the past year, seeking psychological support/intervention due to work pressure in the past month, and nursing adverse events in the past year. All indicators were selected based on the theoretical framework of the Job Demands-Resources (JD-R) model and previous peer studies on nurses’ structural empowerment and occupational stress (15, 16). This questionnaire consists of self-designed single-choice items, and no standardized validated scales were adopted for these baseline demographic and occupational variables.

#### 2.3.2 Conditions for work effectiveness questionnaire-II

The Chinese version of CWEQ-II, developed by Laschinger (17) and localized by Jia (18), was used. It contains 19 items across 6 dimensions: opportunity, information, support, resources, formal empowerment, and informal empowerment. All items are rated on a 5-point Likert scale, with scores ranging from 1 (not at all) to 5 (a great deal). The total score of the scale ranges from 19 to 95. The Chinese version has a content validity index of 0.980 and a Cronbach’s *α* coefficient of 0.904, indicating excellent internal consistency of the scale in this sample and showing good reliability and validity. It has been widely used to assess structural empowerment among Chinese nurses, with higher scores indicating higher levels of structural empowerment.

#### 2.3.3 Perceived stress scale

The Chinese version of the Perceived Stress Scale, developed by Cohen et al. (19) and localized by Yang et al. (20) was adopted. It is a unidimensional scale with 10 items, rated on a 5-point Likert scale (0 = never, 4 = always), with a total score ranging from 0 to 40. The Cronbach’s *α* coefficient of the Chinese version is 0.760. Higher scores indicate higher perceived stress levels.

### 2.4 Data collection

Electronic questionnaires were distributed via Questionnaire Star (Wenjuanxing), a widely used online questionnaire platform in China. Information registration and invitation for the survey were facilitated through the hospital nursing network. To ensure privacy and eliminate situational pressure, all participants accessed the online questionnaire via their personal mobile devices and completed it independently during their off-duty hours or scheduled work breaks. Before the survey, trained researchers provided a standardized explanation of the study’s purpose and significance to all participants. The principles of voluntary participation, anonymity, confidentiality, and informed consent were adopted, and they were informed that there were no right or wrong answers. To ensure data quality, the Wenjuanxing software was designed in such a way that all questions would be answered, each IP address would be allowed to submit only one survey, and the time taken to fill out the form would be documented. Surveys that took less than 3 min to complete or contained responses with an identical pattern regardless of whether they were positively or negatively coded were not used for analysis. A total of 523 questionnaires were distributed, and 5 invalid responses (completed in less than 3 min or with identical repetitive answers) were excluded, resulting in 518 valid questionnaires. This high rate is attributed to the hospital’s favorable atmosphere for nursing research, internal outreach conducted by clinical nursing staff, and the concise, time-efficient questionnaire design, which greatly motivated nurses’ voluntary willingness to participate. Throughout the entire data collection process, participation remained completely voluntary, and no administrative pressure was exerted on any nurses.

### 2.5 Ethical approval

The research protocol was reviewed and approved by the Ethics Committee of Inner Mongolia Medical University Affiliated Hospital (Approval Number: KY2026055). All participants completed the informed consent process electronically before data collection.

### 2.6 Data analysis

SPSS 27.0 and Mplus8.3 software were used for data processing. Mplus8.3 software was used to establish and analyze latent profile models. Three indicators were used to evaluate the model fit: information indicators including Akaike Information Criterion (AIC), Bayesian Information Criterion (BIC), and Sample-Size Adjusted BIC (aBIC), with smaller values indicating better model fit; Entropy was used to assess classification accuracy, ranging from 0 to 1, with larger values indicating more accurate classification (21); and the Lo–Mendell–Rubin Likelihood Ratio Test (LMRT) and Bootstrapped Likelihood Ratio Test (BLRT), where a significant result (*p* < 0.05) indicates that the model with k categories is superior to the model with *k*-1 categories (22). Model estimation employed the maximum likelihood with robust standard errors (MLR) estimator, with five hundred random starting values and one hundred final-stage optimizations specified to safeguard against convergence on local maxima (23). Stable solutions were confirmed by replication of the best loglikelihood across multiple starts, and average posterior probabilities of class membership exceeding 0.80 were treated as evidence of adequate class separation (9). Pairwise binary logistic regression was performed with the low-empowerment profile as the common reference category to compare each of the other three profiles against the low empowerment group. This approach was chosen to facilitate clear interpretation of subgroup-specific contrasts. The presence of common method bias was further evaluated using Harman’s single-factor test (24). SPSS 27.0 software was used for statistical analysis. Quantitative data with a normal distribution were expressed as mean ± standard deviation, and qualitative data were expressed as frequencies and percentages (%). Chi-square tests or F tests were used for univariate intergroup comparisons; Logistic regression analysis was used for multivariate analysis. A two-tailed *p* value <0.05 was considered statistically significant. All statistical analyses in this study were reviewed and verified by a professional biostatistician.

## 3 Results

A total of 523 questionnaires were distributed, with 518 valid questionnaires returned, yielding an effective response rate of 99.04%. Harman’s single-factor test conducted on the combined item pool extracted a first unrotated factor accounting for 28.4% of the total variance, below the 40% threshold conventionally used to indicate substantive common method bias.

### 3.1 Fitting results of latent profile analysis of nurses’ structural empowerment

The mean score of structural empowerment among clinical nurses was (43.76 ± 12.93), and the mean score of perceived stress was (26.83 ± 7.92). Latent profile models with 1 to 5 categories were fitted for structural empowerment among clinical nurses. As the number of categories increased, AIC, BIC, and aBIC values gradually decreased. The 4-class model had the lowest AIC (5979.282), BIC (6119.531), and aBIC (6014.783); the entropy value was 0.996, close to 1, indicating high classification accuracy; the average posterior probability of class membership ranged from 0.987 to 0.998 across the four classes, further supporting clear class separation; both LMRT and BLRT were *p* < 0.0001, suggesting that the 4-class model was significantly better than the 3-class model. Although the five-class model showed lower information criteria, its LMRT was not significant (*p* = 0.0964), indicating that adding a fifth class did not significantly improve fit. In contrast, the four-class model had significant LMRT and BLRT (both *p* < 0.0001), higher parsimony, and clearer theoretical interpretability, and was therefore selected as the optimal model. The detailed results are presented in Table 1. Class 1 (low structural empowerment group) maintained persistently low scores across all six dimensions, which may indicate that nurses in this group suffer insufficient access to information, workplace support, material resources and career development opportunities, alongside limited channels to communicate with management and participate in departmental decision-making. Class 2 (moderate-low structural empowerment group) yielded relatively low scores in the support and resources dimension yet moderate-to-high scores in formal and informal empowerment dimensions. This pattern suggests that nurses in this group enjoy frequent opportunities to communicate with administrators and engage in departmental decision-making. Class 3 (moderate structural empowerment group) obtained high scores in the support and resources dimension but remained low in formal and informal empowerment dimensions. This result reflects that although nurses in this group are equipped with adequate clinical supplies, they have restricted chances to take part in department-level decision-making processes. Class 4 (high structural empowerment group) achieved the highest and evenly distributed scores on all six dimensions. This finding implies that nurses categorized into this group can readily obtain information, workplace assistance, material resources and favorable career advancement opportunities; additionally, they possess ample access to communicate with management personnel and deeply participate in all types of departmental decision-making.

**Table 1:** Fitting results of the latent profile model.

Category	AIC	BIC	aBIC	Entropy	LMRT (*p*)	BLRT (*p*)	Category probability	Number of categories
1	8,499.004	8,550.003	8,511.913	–	–	–	1.000	518
2	7,094.704	7,175.453	7,115.143	0.996	<0.0001	<0.0001	0.780/0.220	404/114
3	6,792.746	6,903.245	6,820.716	0.989	0.0029	<0.0001	0.100/0.666/0.234	52/345/121
**4**	**5,979.282**	**6,119.531**	**6,014.783**	**0.996**	**<0.0001**	**<0.0001**	**0.666/0.102/0.114/0.118**	**345/53/59/61**
5	5,810.586	5,980.585	5,853.617	0.989	0.0964	<0.0001	0.070/0.597/0.102/0.114/0.118	36/309/53/59/61

AIC = Akaike information criterion, BIC = Bayesian information criterion, aBIC = sample-size adjusted BIC, LMRT = Lo–Mendell–Rubin likelihood ratio test, BLRT = bootstrapped likelihood ratio test. Bold values represent the optimal indices of the 4-class latent profile model in Table 1.

**Figure 1:**
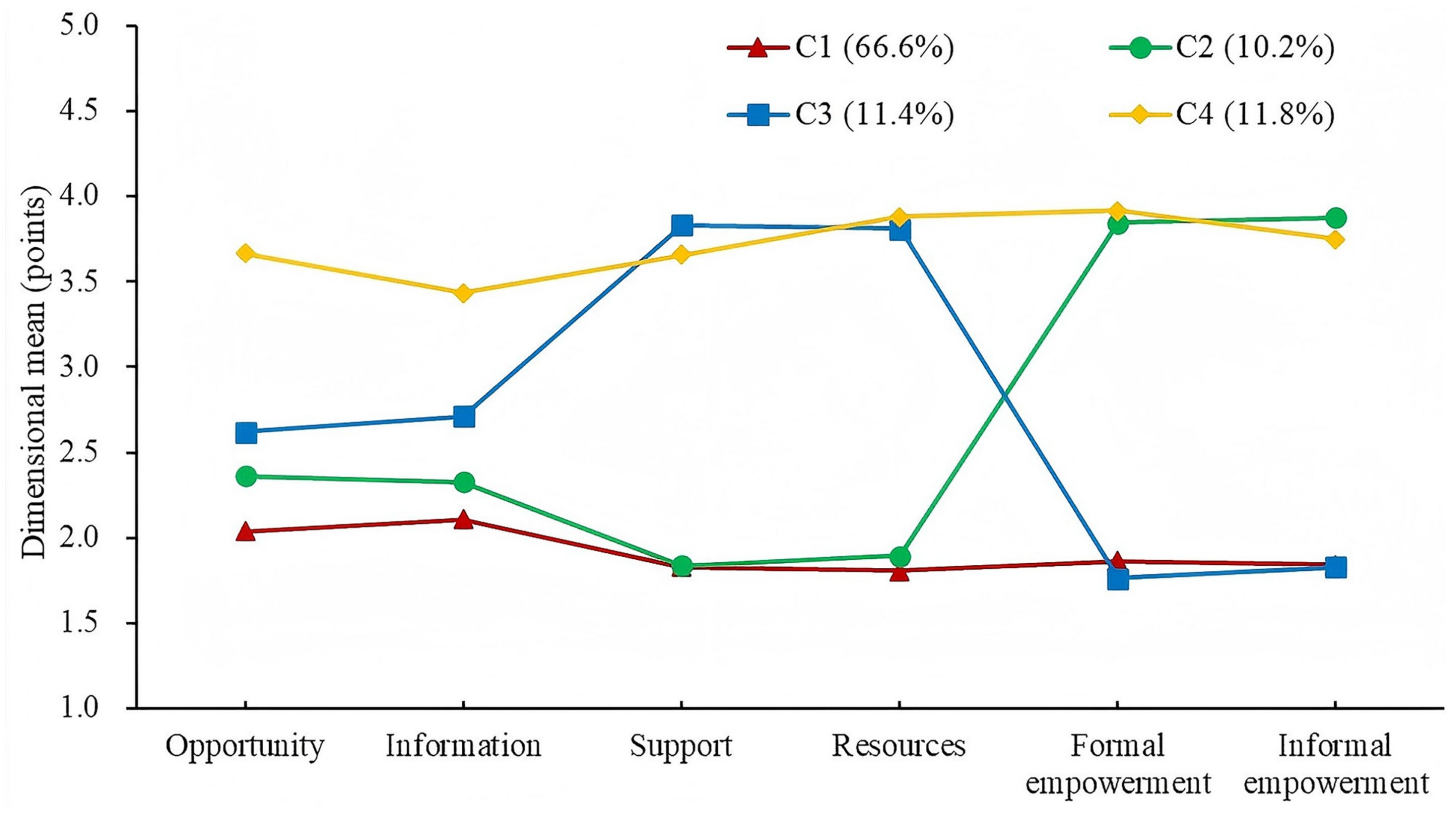
Distribution characteristics of four latent structural profiles across six dimensions.

The distribution characteristics of the four latent categories were as follows:

- Class 1 (*n* = 345, 66.6%): defined as the low structural empowerment group.
- Class 2 (*n* = 53, 10.2%): defined as the moderate-low structural empowerment group.
- Class 3 (*n* = 59, 11.4%): defined as the moderate structural empowerment group.
- Class 4 (*n* = 61, 11.8%): defined as the high structural empowerment group (Figure 1).

### 3.2 Univariate analysis of nurses’ structural empowerment

Univariate analysis showed that age, marital status, highest educational background, position, employment method, work experience, average monthly income, monthly night shift frequency in the past year, sleep status, self-rated health status, seeking psychological support/intervention in the past month, involvement in or occurrence of nursing adverse events in the past year, and perceived stress were influencing factors of structural empowerment categories (*p* < 0.05). See Table 2 for details.

**Table 2:** Univariate analysis of latent structural empowerment categories [*n* = 518, *n* (%)].

Item	Number	C1 (*n* = 345)	C2 (*n* = 53)	C3 (*n* = 59)	C4 (*n* = 61)	*χ^2^*/*F*	*p*
Gender						2.205^a^	0.531
Men	42	25 (7.25)	7 (13.21)	5 (8.47)	5 (8.20)		
Women	476	320 (92.75)	46 (86.79)	54 (91.53)	56 (91.80)		
Age (years)						26.916^a^	0.001
≤25	147	89 (25.80)	13 (24.53)	20 (33.90)	25 (40.98)		
26–35	236	150 (43.48)	29 (54.72)	28 (47.46)	29 (47.54)		
36–45	100	81 (23.48)	10 (18.87)	9 (15.25)	0 (0.00)		
>45	35	25 (7.25)	1 (1.89)	2 (3.39)	7 (11.48)		
Marital status						13.627^a^	0.034
Unmarried	101	67 (19.42)	5 (9.43)	10 (16.95)	19 (31.15)		
Married	375	253 (73.33)	45 (84.91)	43 (72.88)	34 (55.74)		
Divorced or widowed	42	25 (7.25)	3 (5.66)	6 (10.17)	8 (13.11)		
Highest educational background						29.475^a^	<0.001
College or below	164	90 (26.09)	17 (32.08)	25 (42.37)	32 (52.46)		
Bachelor’s degree	264	180 (52.17)	27 (50.94)	29 (49.15)	28 (45.90)		
Master’s degree or above	90	75 (21.74)	9 (16.98)	5 (8.47)	1 (1.64)		
Professional title						1.507^a^	0.959
Nurse	471	315 (91.30)	49 (92.45)	53 (89.83)	54 (88.52)		
Senior nurse	36	23 (6.67)	3 (5.66)	4 (6.78)	6 (9.84)		
Nurse practitioner or above	11	7 (2.03)	1 (1.89)	2 (3.39)	1 (1.64)		
Position						13.172^a^	0.004
Nurse	493	320 (92.75)	53 (100.00)	59 (100.00)	61 (100.00)		
Nursing manager	25	25 (7.25)	0 (0.00)	0 (0.00)	0 (0.00)		
Employment method						13.841^a^	0.031
Contract	262	172 (49.86)	30 (56.60)	33 (55.93)	27 (44.26)		
Staff establishment	192	124 (35.94)	16 (30.19)	19 (32.20)	33 (54.10)		
Others	64	49 (14.20)	7 (13.21)	7 (11.86)	1 (1.64)		
Work experience (years)						27.708^a^	0.006
≤5	220	137 (39.71)	25 (47.17)	26 (44.07)	32 (52.46)		
6–10	133	89 (25.80)	13 (24.53)	17 (28.81)	14 (22.95)		
11–15	103	61 (17.68)	14 (26.42)	13 (22.03)	15 (24.59)		
16–20	51	49 (14.20)	0 (0.00)	2 (3.39)	0 (0.00)		
>20	11	9 (2.61)	1 (1.89)	1 (1.69)	0 (0.00)		
Department						27.196^a^	0.164
Internal medicine department	75	47 (13.62)	9 (16.98)	5 (8.47)	14 (22.95)		
Surgery department	59	41 (11.88)	3 (5.66)	8 (13.56)	7 (11.48)		
Gynecology department	85	65 (18.84)	3 (5.66)	8 (13.56)	9 (14.75)		
Paediatrics department	109	72 (20.87)	11 (20.75)	17 (28.81)	9 (14.75)		
Emergency department	25	18 (5.22)	4 (7.55)	0 (0.00)	3 (4.92)		
ICU	43	27 (7.83)	7 (13.21)	4 (6.78)	5 (8.20)		
Operating room	111	68 (19.71)	13 (24.53)	17 (28.81)	13 (21.31)		
Others	11	7 (2.03)	3 (5.66)	0 (0.00)	1 (1.64)		
Average monthly income (yuan)						32.374^a^	<0.001
≤3,999	143	80 (23.19)	15 (28.30)	26 (44.07)	22 (36.07)		
4,000–5,999	237	153 (44.35)	28 (52.83)	27 (45.76)	29 (47.54)		
6,000–7,999	103	77 (22.32)	10 (18.87)	6 (10.17)	10 (16.39)		
≥8,000	35	35 (10.14)	0 (0.00)	0 (0.00)	0 (0.00)		
Monthly night shift frequency in the past year						28.686^a^	0.001
No	112	65 (18.84)	19 (35.85)	13 (22.03)	15 (24.59)		
1–4	252	164 (47.54)	21 (39.62)	33 (55.93)	34 (55.74)		
5–8	104	68 (19.71)	12 (22.64)	13 (22.03)	11 (18.03)		
≥9	50	48 (13.91)	1 (1.89)	0 (0.00)	1 (1.64)		
Average daily working hours (hour)						11.770^a^	0.227
≤8	158	108 (31.30)	18 (33.96)	18 (30.51)	14 (22.95)		
9–10	228	150 (43.48)	18 (33.96)	26 (44.07)	34 (55.74)		
11–12	98	63 (18.26)	10 (18.87)	13 (22.03)	12 (19.67)		
>12	34	24 (6.96)	7 (13.21)	2 (3.39)	1 (1.64)		
Sleep status						25.709^a^	<0.001
Normal	288	183 (53.04)	23 (43.40)	39 (66.10)	43 (70.49)		
Insomnia	143	89 (25.80)	20 (37.74)	16 (27.12)	18 (29.51)		
Other conditions	87	73 (21.16)	10 (18.87)	4 (6.78)	0 (0.00)		
Self-rated health status						39.938^a^	<0.001
Good	153	80 (23.19)	20 (37.74)	23 (38.98)	30 (49.18)		
General	250	165 (47.83)	28 (52.83)	27 (45.76)	30 (49.18)		
Poor	115	100 (28.99)	5 (9.43)	9 (15.25)	1 (1.64)		
Experience of verbal or physical violence from patients/families in the past year						2.138^a^	0.544
Yes	191	121 (35.07)	19 (35.85)	25 (42.37)	26 (42.62)		
No	327	224 (64.93)	34 (64.15)	34 (57.63)	35 (57.38)		
Seeking psychological support/intervention in the past month						28.969^a^	<0.001
Yes	355	220 (63.77)	36 (67.92)	39 (66.10)	60 (98.36)		
No	163	125 (36.23)	17 (32.08)	20 (33.90)	1 (1.64)		
Involvement in or occurrence of nursing adverse events in the past year						27.392^a^	<0.001
Yes	228	127 (36.81)	27 (50.94)	31 (52.54)	43 (70.49)		
No	290	218 (63.19)	26 (49.06)	28 (47.46)	18 (29.51)		
Perceived stress (score, x ± s)	518	29.14 ± 5.77	26.43 ± 8.91	25.53 ± 8.19	15.36 ± 6.99	76.213^b^	<0.001

^a^ represents the *χ*^2^ value, ^b^ represents the *F* value; C1 = low structural empowerment group, C2 = moderate-low structural empowerment group, C3 = moderate structural empowerment group, C4 = high structural empowerment group.

### 3.3 Multivariate analysis of nurses’ structural empowerment categories

Multivariate Logistic regression analysis was performed with latent categories of structural empowerment as the dependent variable (low structural empowerment group as reference) and statistically significant variables in univariate analysis as independent variables, the values assigned to the independent variables are shown in Table 3. Results showed that monthly night shift frequency in the past year, self-rated health status, involvement in or occurrence of nursing adverse events in the past year, and perceived stress were core influencing factors of latent profiles of structural empowerment among clinical nurses (*p* < 0.05). See Table 4 for details.

**Table 3:** Method of variable assignment.

Independent variable	Assignment
Age (years)	≤25 = 1, 26–35 = 2, 36–45 = 3, >45 = 4
Marital status	Unmarried = 1, Married = 2, Divorced or widowed = 3
Highest educational background	College or Below = 1, Bachelor’s Degree = 2, Master’s Degree or Above = 3
Position	Nurse = 1, Nursing manager = 2
Employment method	Contract = 1, Staff Establishment = 2, Others = 3
Work experience (years)	≤5 = 1, 6–10 = 2, 11–15 = 3, 16–20 = 4, >20 = 5
Average monthly income (yuan)	≤3,999 = 1, 4,000–5,999 = 2, 6,000–7,999 = 3, ≥8,000 = 4
Monthly night shift frequency in the past year	No = 1, 1–4 = 2, 5–8 = 3, ≥9 = 4
Sleep status	Normal = 1, Insomnia = 2, Other conditions = 3
Self-rated health status	Good = 1, General = 2, Poor = 3
Seeking psychological support/intervention in the past month	Yes = 1, No = 2
Involvement in or occurrence of nursing adverse events in the past year	Yes = 1, No = 2
Perceived stress	Original value input

**Table 4:** Multivariate analysis results (*n* = 518).

Item	*β*	SE	Wald *χ*^2^	*p*	OR (95%CI)
C2 vs. C1					
Monthly night shift frequency in the past year (no)	2.173	1.080	4.045	0.044	8.781 (1.057–72.964)
Self-rated health status (good)	1.445	0.567	6.503	0.011	4.243 (1.397–12.886)
Self-rated health status (general)	1.119	0.538	4.326	0.038	3.061 (1.067–8.783)
C3 vs. C1					
Perceived stress	−0.050	0.021	5.670	0.017	0.951 (0.913–0.991)
C4 vs. C1					
Perceived stress	−0.175	0.029	36.113	<0.001	0.840 (0.793–0.889)
Self-rated health status (good)	3.007	1.211	6.165	0.013	20.233 (1.884–217.291)
Self-rated health status (general)	3.062	1.242	6.081	0.014	21.367 (1.874–243.581)
Involvement in or occurrence of nursing adverse events in the past year (yes)	1.149	0.438	6.882	0.009	3.155 (1.337–7.446)

Use the low-structure empowerment group (C1) as the reference.

## 4 Discussion

### 4.1 Structural empowerment among clinical nurses is at a low level with significant latent category heterogeneity

The structural empowerment score of clinical nurses was at a moderately low level, consistent with findings by Ibrahim (25) and Ta′an et al. (26), and lower than those by Wang (27) and Liu et al. (28) Clinical nurses in this tertiary hospital showed relatively low structural empowerment, indicating obvious shortcomings in information supply, resource guarantee, career opportunities, management support, and empowerment in current nursing organizations. Structural empowerment, as a core occupational resource for nursing, has not been fully released. Managers should further attach importance to insufficient empowerment among clinical nurses and optimize the empowerment mechanism of clinical nursing management (29).

Our four-profile solution revealed substantial heterogeneity in structural empowerment, with the low empowerment group constituting the largest subgroup. The present findings are consistent with the realities of the nursing profession in recent years, which are characterized by workforce shortages and heavy workloads (30, 31). Against the backdrop of widespread nursing workforce shortages and heavy workloads, clinical nurses exhibit low perceptions of structural empowerment (32, 33). Meanwhile, discrepancies in working scenarios and professional states among nurses create differing perceptions of organizational empowerment resources, ultimately forming the observed group heterogeneity of structural empowerment.

The proportion of each subgroup further indicates that most clinical nurses have not yet obtained sufficient structural empowerment, and the characteristics of different empowerment-level groups are significantly different with prominent heterogeneity. Healthcare managers should not adopt a homogeneous management strategy, but formulate stratified and classified empowerment intervention plans according to the characteristics of different latent categories.

### 4.2 Analysis of influencing factors of latent profiles of structural empowerment among clinical nurses

#### 4.2.1 Monthly night shift frequency in the past year

Multivariate Logistic regression showed that nurses with lower night shift frequency were more likely to obtain higher structural empowerment. This phenomenon may be attributed to the following reasons, nurses with lower night shift frequency have a more stable work rhythm and more career development opportunities (34), and are more likely to obtain information support and resource allocation authority, resulting in a higher level of structural empowerment (35).

The results suggest that healthcare managers should prioritize reducing night shift frequency for nurses in the low structural empowerment group, ensure regular work-rest schedules, help them maintain a stable work rhythm, and reserve more time for professional development activities such as departmental training and management communication, so as to gradually improve their access to information support and resource allocation and enhance their structural empowerment (36). For the moderate-low and moderate structural empowerment groups, flexible scheduling can be adopted to avoid consecutive night shifts, and sufficient rest time should be provided after night shifts (37). Meanwhile, these nurses should be encouraged and guaranteed to equally participate in departmental resource allocation discussions and process optimization meetings during non-night-shift periods, so that they can have more say in clinical decision-making and gradually improve their perception of structural empowerment (38). For the high structural empowerment group, the existing night shift arrangement can be maintained (39), while channels for nurses to obtain departmental information and apply for work resources should be further unblocked to consolidate the existing high level of structural empowerment, and guide nurses to actively participate in departmental management and construction, so as to exert the positive effect of high structural empowerment on nurses’ professional growth.

#### 4.2.2 Self-rated health status

Nurses with good or average self-rated health status had a higher probability of being in the high structural empowerment group, indicating that the better the health status of clinical nurses, the higher the level of structural empowerment (40). Good health is the foundation for nurses to fulfill their job responsibilities, access organizational resources, and participate in decision-making (41). Nurses with poor health are prone to insufficient energy, occupational burnout, work withdrawal and other problems, making it difficult to actively strive for information, support and development opportunities, and their ability to perceive and utilize empowerment decreases.

Healthcare managers should implement targeted interventions for different groups: for the low structural empowerment group, nurses’ health management can be integrated into the structural empowerment support system so as to help them gradually recover their health, regain the basic ability to access organizational resources and participate in work decision-making, and further improve their structural empowerment. For nurses in the moderate-low and moderate structural empowerment groups, in addition to routine health management, stress management and psychological resilience training should be added to help them effectively cope with setbacks and high-pressure situations in clinical work and maintain a good physical and mental state, so as to support their further development towards a high empowerment level (42). For nurses in the high structural empowerment group, regular health follow-ups and personalized health plans can be adopted to maintain their good health status, help nurses maintain sufficient energy to participate in work and actively access various organizational resources, so as to further consolidate and improve their structural empowerment level (43).

#### 4.2.3 Involvement in or occurrence of nursing adverse events in the past year

This study found that nurses who had experienced nursing adverse events were more likely to be classified into the high structural empowerment group, which differs from conventional understanding. A possible explanation is that after involvement in or occurrence of nursing adverse events, nurses fully learn from the experience, participate deeply in root-cause analysis and process optimization of adverse events, and improve their knowledge of nursing adverse events, thereby further enhancing their perception of organizational structural empowerment. For nurses in the low structural empowerment group, nursing leaders can provide proactive one-on-one support and guidance to help them conduct correct attribution, and gradually invite them to attend case conferences on adverse events to deepen their understanding, so that they can gradually gain access to information, resources, and decision-making power (44). For nurses in the high structural empowerment group, nursing leaders should deliver systematic support to all nurses involved in adverse events, establish a clear non-punitive reporting system and psychological support mechanism, and ensure that nurses receive guidance and assistance after reporting adverse events (45).

#### 4.2.4 Perceived stress as the core negative predictor of structural empowerment

This study suggests an association where higher perceived stress relates to lower perceived empowerment. However, due to the cross-sectional design of this study, we cannot determine the causal direction of this correlation. According to the JD-R theory, under high pressure, nurses’ cognitive resources are heavily occupied, making it difficult to effectively utilize the empowerment resources provided by the organization (46, 47). They cannot actively strive for organizational resource support, and their perception of their own empowerment will also decrease. Therefore, a high stress level not only directly damages nurses’ physical and mental health, but also indirectly affects their work efficiency and professional development by inhibiting their perception and utilization of structural empowerment (48).

For nurses in the low structural empowerment group, nursing leaders should accurately identify their stressors through questionnaires, interviews and other methods, reduce workload by optimizing staffing, simplifying document processes and introducing auxiliary tools, establish open communication channels to respond to their demands in a timely manner, and enhance their sense of control (49). For nurses in the moderate-low and moderate structural empowerment groups, while reducing their pressure, emphasis should be placed on providing empowerment as a stress buffer resource, granting them the autonomy to adjust work methods within a certain range, allowing them to participate in scheduling discussions, and increasing their sense of control over work, so as to break the stress cycle (50). For nurses in the high structural empowerment group, the management focus is to maintain this benign state, ensure that they continue to have sufficient resources and support to cope with challenges, and avoid pressure rebound caused by resource deprivation.

## 5 Conclusion

This study identified four latent categories of structural empowerment among clinical nurses, demonstrating significant population heterogeneity. Monthly night shift frequency in the past year, self-rated health status, involvement in or occurrence of nursing adverse events in the past year, and perceived stress are core influencing factors. These findings suggest that targeted occupational health interventions, such as optimized shift scheduling, stress reduction, health promotion, and a learning-oriented safety culture may help enhance structural empowerment and support workforce stability. Future intervention studies are needed to confirm these effects. The findings may inform future occupational health strategies.

## 6 Limitations

This study has several limitations. First, the cross-sectional design prevents causal inferences between structural empowerment profiles and occupational outcomes. However, the findings provide critical baseline data on empowerment heterogeneity and its correlates, offering a robust foundation for future longitudinal and intervention studies in occupational health. Second, convenience sampling from a single center may limit generalizability to other healthcare settings, but the large sample size (*n* = 518) and rigorous statistical analysis enhance internal validity, supporting the relevance of these results to similar acute care environments.

While the support from clinical staff and user-friendly questionnaire design brought about a high participation rate, we acknowledge that the extremely high response rate may introduce potential social desirability bias. Future multi-center investigations will help verify our findings.

## Data Availability

The raw data supporting the conclusions of this article will be made available by the authors, without undue reservation.

## Footnotes

1
www.wjx.cn
